# Supplementary material for: Vestigial auriculomotor activity indicates the direction of auditory attention in humans
Source: eLife. 2020 Jul 3;9:e54536. doi: 10.7554/eLife.54536 (PMC7334025; doi:10.7554/eLife.54536)
Supplement: Supplementary file 1. [file elife-54536-supp1.pdf]

Supplementary File 1

The following tables summarize the statistical results when using gaze shifts and the neck muscle activity additionally for the artifact rejection in the individual muscle electromyograms. The results demonstrate that the inclusion of this information has no major impact, strengthening the conclusions made from `Figure 8`.

Full Signal									
Artifact rejection based on channel only					Artifact rejection based on channel, M.SCM and HEOG				
Source	Measure	F	Sig.	Partial Eta Squared	Source	Measure	F	Sig.	Partial Eta Squared
ipsiCont	PAM	14.792	0.001	0.438	ipsiCont	PAM	14.748	0.001	0.437
	AAM	4.717	0.043	0.199		AAM	5.359	0.032	0.220
	TAM	0.072	0.791	0.004		TAM	0.058	0.813	0.003
	SAM	19.626	0.000	0.508		SAM	20.306	0.000	0.517
ipsiCont * ageGroup	PAM	0.049	0.828	0.003	ipsiCont * ageGroup	PAM	0.058	0.812	0.003
	AAM	0.002	0.961	0.000		AAM	0.008	0.928	0.000
	TAM	0.018	0.896	0.001		TAM	0.023	0.880	0.001
	SAM	1.172	0.292	0.058		SAM	1.530	0.231	0.075
anteriority	PAM	5.760	0.027	0.233	anteriority	PAM	5.811	0.026	0.234
	AAM	3.167	0.091	0.143		AAM	2.882	0.106	0.132
	TAM	8.137	0.010	0.300		TAM	7.635	0.012	0.287
	SAM	12.094	0.003	0.389		SAM	11.574	0.003	0.379
anteriority * ageGroup	PAM	0.284	0.600	0.015	anteriority * ageGroup	PAM	0.348	0.562	0.018
	AAM	0.010	0.923	0.001		AAM	0.039	0.846	0.002
	TAM	1.353	0.259	0.066		TAM	1.158	0.295	0.057
ipsiCont * anteriority	PAM	3.507	0.077	0.156	ipsiCont * anteriority	PAM	3.730	0.069	0.164
	AAM	2.187	0.156	0.103		AAM	2.961	0.102	0.135
	TAM	0.492	0.492	0.025		TAM	0.529	0.476	0.027
	SAM	0.099	0.757	0.005		SAM	0.225	0.641	0.012
ipsiCont * anteriority * ageGroup	PAM	0.173	0.682	0.009	ipsiCont * anteriority * ageGroup	PAM	0.193	0.666	0.010
	AAM	0.001	0.981	0.000		AAM	0.036	0.852	0.002
	TAM	0.314	0.582	0.016		TAM	0.282	0.601	0.015
	SAM	0.047	0.831	0.002		SAM	0.151	0.701	0.008

Table 1: `Experiment 2 - table supplement 1:` Results of the repeated measures ANOVA with three within-subjects factors for all four auricular muscles. ipsiCont: ipsilateral vs. contralateral channel (with respect to the attended side). ageGroup: older vs. younger adults. anteriority: stimulus presentation from the front or back speakers. The left columns show the results when only the corresponding channel was used to reject artifacts, while the right columns display the results when artifact rejection additionally considers the M.SCM and HEOG.

Subbands PAM									
Artifact rejection based on channel only					Artifact rejection based on channel, M.SCM and HEOG				
Source	Measure	F	Sig.	Partial Eta Squared	Source	Measure	F	Sig.	Partial Eta Squared
ipsiCont	f1	8.492	0.009	0.309	ipsiCont	f1	8.282	0.010	0.304
	f2	10.668	0.004	0.360		f2	10.735	0.004	0.361
	f3	12.544	0.002	0.398		f3	12.282	0.002	0.393
	f4	14.650	0.001	0.435		f4	14.413	0.001	0.431
	f5	17.185	0.001	0.475		f5	17.086	0.001	0.473
	f6	16.865	0.001	0.470		f6	17.133	0.001	0.474
	f7	17.671	0.000	0.482		f7	18.116	0.000	0.488
	f8	10.320	0.005	0.352		f8	10.432	0.004	0.354
ipsiCont * ageGroup	f1	0.125	0.728	0.007	ipsiCont * ageGroup	f1	0.158	0.695	0.008
	f2	0.368	0.551	0.019		f2	0.418	0.525	0.022
	f3	0.077	0.785	0.004		f3	0.107	0.747	0.006
	f4	0.003	0.959	0.000		f4	0.001	0.982	0.000
	f5	0.000	0.985	0.000		f5	0.000	0.992	0.000
	f6	0.013	0.911	0.001		f6	0.013	0.909	0.001
	f7	0.004	0.950	0.000		f7	0.007	0.936	0.000
	f8	0.000	0.985	0.000		f8	0.003	0.958	0.000
anteriority	f1	2.409	0.137	0.113	anteriority	f1	2.430	0.136	0.113
	f2	4.083	0.058	0.177		f2	4.059	0.058	0.176
	f3	6.217	0.022	0.247		f3	6.269	0.022	0.248
	f4	6.403	0.020	0.252		f4	6.521	0.019	0.256
	f5	6.260	0.022	0.248		f5	6.362	0.021	0.251
	f6	5.275	0.033	0.217		f6	5.289	0.033	0.218
	f7	4.308	0.052	0.185		f7	4.349	0.051	0.186
	f8	11.086	0.004	0.368		f8	11.072	0.004	0.368
anteriority * ageGroup	f1	1.983	0.175	0.095	anteriority * ageGroup	f1	2.097	0.164	0.099
	f2	1.137	0.300	0.056		f2	1.350	0.260	0.066
	f3	0.109	0.745	0.006		f3	0.144	0.709	0.008
	f4	0.056	0.816	0.003		f4	0.069	0.796	0.004
	f5	0.176	0.679	0.009		f5	0.189	0.669	0.010
	f6	0.680	0.420	0.035		f6	0.724	0.405	0.037
	f7	0.950	0.342	0.048		f7	0.994	0.331	0.050
	f8	0.685	0.418	0.035		f8	0.673	0.422	0.034
ipsiCont * anteriority	f1	0.345	0.564	0.018	ipsiCont * anteriority	f1	0.469	0.502	0.024
	f2	3.362	0.082	0.150		f2	3.932	0.062	0.171
	f3	6.342	0.021	0.250		f3	6.733	0.018	0.262
	f4	4.485	0.048	0.191		f4	4.344	0.051	0.186
	f5	3.130	0.093	0.141		f5	3.088	0.095	0.140
	f6	2.720	0.116	0.125		f6	2.779	0.112	0.128
	f7	1.988	0.175	0.095		f7	1.949	0.179	0.093
	f8	0.077	0.785	0.004		f8	0.070	0.794	0.004
ipsiCont * anteriority * ageGroup	f1	0.340	0.567	0.018	ipsiCont * anteriority * ageGroup	f1	0.380	0.545	0.020
	f2	1.025	0.324	0.051		f2	1.070	0.314	0.053
	f3	0.325	0.575	0.017		f3	0.342	0.565	0.018
	f4	0.174	0.681	0.009		f4	0.164	0.690	0.009
	f5	1.117	0.304	0.056		f5	1.126	0.302	0.056
	f6	1.543	0.229	0.075		f6	1.608	0.220	0.078
	f7	2.691	0.117	0.124		f7	2.702	0.117	0.125
	f8	0.902	0.354	0.045		f8	0.907	0.353	0.046

Table 2: `Experiment 2 - table supplement 2:` Results of the repeated measures ANOVA with three within-subjects factors for the frequency decomposed PAM. f1 to f8 refer to the subbands in descending order of frequency. ipsiCont: ipsilateral vs. contralateral channel (with respect to the attended side). ageGroup: older vs. younger adults. anteriority: stimulus presentation from the front or back speakers. The left columns show the results when only the corresponding channel was used to reject artifacts, while the right columns display the results when artifact rejection additionally considers the M.SCM and HEOG.

Subbands AAM									
Artifact rejection based on channel only					Artifact rejection based on channel, M.SCM and HEOG				
Source	Measure	F	Sig.	Partial Eta Squared	Source	Measure	F	Sig.	Partial Eta Squared
ipsiCont	f1	0.900	0.355	0.045	ipsiCont	f1	1.277	0.273	0.063
	f2	1.870	0.187	0.090		f2	2.311	0.145	0.108
	f3	3.594	0.073	0.159		f3	4.020	0.059	0.175
	f4	7.114	0.015	0.272		f4	7.664	0.012	0.287
	f5	8.231	0.010	0.302		f5	8.488	0.009	0.309
	f6	6.789	0.017	0.263		f6	7.065	0.016	0.271
	f7	4.333	0.051	0.186		f7	4.293	0.052	0.184
	f8	5.408	0.031	0.222		f8	5.851	0.026	0.235
ipsiCont * ageGroup	f1	0.001	0.971	0.000	ipsiCont * ageGroup	f1	0.015	0.904	0.001
	f2	0.228	0.639	0.012		f2	0.434	0.518	0.022
	f3	0.043	0.837	0.002		f3	0.146	0.707	0.008
	f4	0.000	0.983	0.000		f4	0.011	0.917	0.001
	f5	0.102	0.752	0.005		f5	0.095	0.762	0.005
	f6	0.707	0.411	0.036		f6	0.719	0.407	0.036
	f7	1.751	0.201	0.084		f7	1.778	0.198	0.086
	f8	0.182	0.674	0.010		f8	0.120	0.733	0.006
anteriority	f1	3.259	0.087	0.146	anteriority	f1	2.831	0.109	0.130
	f2	3.080	0.095	0.139		f2	2.694	0.117	0.124
	f3	3.828	0.065	0.168		f3	3.526	0.076	0.157
	f4	4.262	0.053	0.183		f4	4.287	0.052	0.184
	f5	3.190	0.090	0.144		f5	2.992	0.100	0.136
	f6	1.795	0.196	0.086		f6	1.592	0.222	0.077
	f7	1.872	0.187	0.090		f7	1.869	0.188	0.090
	f8	19.224	0.000	0.503		f8	19.582	0.000	0.508
anteriority * ageGroup	f1	0.112	0.742	0.006	anteriority * ageGroup	f1	0.214	0.649	0.011
	f2	0.005	0.944	0.000		f2	0.052	0.822	0.003
	f3	0.012	0.916	0.001		f3	0.038	0.847	0.002
	f4	0.051	0.824	0.003		f4	0.054	0.819	0.003
	f5	0.001	0.970	0.000		f5	0.001	0.980	0.000
	f6	0.032	0.860	0.002		f6	0.010	0.921	0.001
	f7	0.002	0.967	0.000		f7	0.001	0.974	0.000
	f8	0.765	0.393	0.039		f8	0.807	0.380	0.041
ipsiCont * anteriority	f1	0.039	0.846	0.002	ipsiCont * anteriority	f1	0.005	0.946	0.000
	f2	0.070	0.794	0.004		f2	0.000	0.997	0.000
	f3	0.508	0.485	0.026		f3	1.018	0.326	0.051
	f4	3.528	0.076	0.157		f4	3.781	0.067	0.166
	f5	4.482	0.048	0.191		f5	4.865	0.040	0.204
	f6	3.661	0.071	0.162		f6	4.322	0.051	0.185
	f7	1.812	0.194	0.087		f7	1.993	0.174	0.095
	f8	0.213	0.649	0.011		f8	0.220	0.644	0.011
ipsiCont * anteriority * ageGroup	f1	1.304	0.268	0.064	ipsiCont * anteriority * ageGroup	f1	1.504	0.235	0.073
	f2	0.192	0.666	0.010		f2	0.100	0.755	0.005
	f3	0.001	0.974	0.000		f3	0.107	0.748	0.006
	f4	0.001	0.981	0.000		f4	0.048	0.829	0.003
	f5	0.037	0.850	0.002		f5	0.112	0.742	0.006
	f6	0.075	0.788	0.004		f6	0.196	0.663	0.010
	f7	0.173	0.682	0.009		f7	0.175	0.680	0.009
	f8	0.286	0.599	0.015		f8	0.282	0.602	0.015

Table 3: `Experiment 2 - table supplement 3:` Results of the repeated measures ANOVA with three within-subjects factors for the frequency decomposed AAM. f1 to f8 refer to the subbands in descending order of frequency. ipsiCont: ipsilateral vs. contralateral channel (with respect to the attended side). ageGroup: older vs. younger adults. anteriority: stimulus presentation from the front or back speakers. The left columns show the results when only the corresponding channel was used to reject artifacts, while the right columns display the results when artifact rejection additionally considers the M.SCM and HEOG.

Subbands TAM									
Artifact rejection based on channel only					Artifact rejection based on channel, M.SCM and HEOG				
Source	Measure	F	Sig.	Partial Eta Squared	Source	Measure	F	Sig.	Partial Eta Squared
ipsiCont	f1	0.006	0.937	0.000	ipsiCont	f1	0.012	0.915	0.001
	f2	0.005	0.946	0.000		f2	0.009	0.927	0.000
	f3	0.064	0.803	0.003		f3	0.054	0.818	0.003
	f4	0.308	0.585	0.016		f4	0.277	0.605	0.014
	f5	0.461	0.505	0.024		f5	0.466	0.503	0.024
	f6	0.558	0.464	0.029		f6	0.571	0.459	0.029
	f7	0.381	0.544	0.020		f7	0.390	0.540	0.020
	f8	1.558	0.227	0.076		f8	1.434	0.246	0.070
ipsiCont * ageGroup	f1	0.220	0.645	0.011	ipsiCont * ageGroup	f1	0.270	0.609	0.014
	f2	0.087	0.771	0.005		f2	0.093	0.763	0.005
	f3	0.004	0.948	0.000		f3	0.008	0.930	0.000
	f4	0.001	0.971	0.000		f4	0.004	0.949	0.000
	f5	0.041	0.842	0.002		f5	0.043	0.838	0.002
	f6	0.133	0.719	0.007		f6	0.146	0.707	0.008
	f7	0.070	0.794	0.004		f7	0.077	0.784	0.004
	f8	0.036	0.851	0.002		f8	0.091	0.767	0.005
anteriority	f1	6.104	0.023	0.243	anteriority	f1	6.063	0.024	0.242
	f2	5.953	0.025	0.239		f2	5.635	0.028	0.229
	f3	7.169	0.015	0.274		f3	6.688	0.018	0.260
	f4	8.129	0.010	0.300		f4	7.834	0.011	0.292
	f5	8.176	0.010	0.301		f5	7.666	0.012	0.287
	f6	7.942	0.011	0.295		f6	7.638	0.012	0.287
	f7	7.330	0.014	0.278		f7	7.287	0.014	0.277
	f8	42.497	0.000	0.691		f8	42.834	0.000	0.693
anteriority * ageGroup	f1	0.377	0.546	0.019	anteriority * ageGroup	f1	0.391	0.539	0.020
	f2	1.138	0.299	0.057		f2	1.012	0.327	0.051
	f3	1.682	0.210	0.081		f3	1.402	0.251	0.069
	f4	1.517	0.233	0.074		f4	1.343	0.261	0.066
	f5	0.793	0.384	0.040		f5	0.637	0.435	0.032
	f6	0.446	0.512	0.023		f6	0.355	0.558	0.018
	f7	0.516	0.481	0.026		f7	0.509	0.484	0.026
	f8	1.709	0.207	0.083		f8	1.688	0.209	0.082
ipsiCont * anteriority	f1	0.170	0.685	0.009	ipsiCont * anteriority	f1	0.206	0.655	0.011
	f2	0.380	0.545	0.020		f2	0.450	0.510	0.023
	f3	0.918	0.350	0.046		f3	1.079	0.312	0.054
	f4	0.533	0.474	0.027		f4	0.598	0.449	0.031
	f5	0.175	0.681	0.009		f5	0.249	0.624	0.013
	f6	0.075	0.787	0.004		f6	0.109	0.745	0.006
	f7	0.658	0.427	0.033		f7	0.735	0.402	0.037
	f8	0.581	0.455	0.030		f8	0.608	0.445	0.031
ipsiCont * anteriority * ageGroup	f1	0.519	0.480	0.027	ipsiCont * anteriority * ageGroup	f1	0.482	0.496	0.025
	f2	0.571	0.459	0.029		f2	0.529	0.476	0.027
	f3	0.428	0.521	0.022		f3	0.341	0.566	0.018
	f4	0.038	0.848	0.002		f4	0.020	0.888	0.001
	f5	0.022	0.884	0.001		f5	0.061	0.808	0.003
	f6	0.011	0.916	0.001		f6	0.038	0.848	0.002
	f7	0.103	0.751	0.005		f7	0.130	0.723	0.007
	f8	0.186	0.671	0.010		f8	0.172	0.683	0.009

Table 4: `Experiment 2 - table supplement 4:` Results of the repeated measures ANOVA with three within-subjects factors for the frequency decomposed TAM. f1 to f8 refer to the subbands in descending order of frequency. ipsiCont: ipsilateral vs. contralateral channel (with respect to the attended side). ageGroup: older vs. younger adults. anteriority: stimulus presentation from the front or back speakers. The left columns show the results when only the corresponding channel was used to reject artifacts, while the right columns display the results when artifact rejection additionally considers the M.SCM and HEOG.

Subbands SAM									
Artifact rejection based on channel only					Artifact rejection based on channel, M.SCM and HEOG				
Source	Measure	F	Sig.	Partial Eta Squared	Source	Measure	F	Sig.	Partial Eta Squared
ipsiCont	f1	10.582	0.004	0.358	ipsiCont	f1	11.524	0.003	0.378
	f2	15.800	0.001	0.454		f2	17.718	0.000	0.483
	f3	16.313	0.001	0.462		f3	16.499	0.001	0.465
	f4	19.565	0.000	0.507		f4	20.312	0.000	0.517
	f5	24.001	0.000	0.558		f5	24.642	0.000	0.565
	f6	23.512	0.000	0.553		f6	23.446	0.000	0.552
	f7	32.793	0.000	0.633		f7	33.528	0.000	0.638
	f8	10.776	0.004	0.362		f8	11.013	0.004	0.367
ipsiCont * ageGroup	f1	0.005	0.944	0.000	ipsiCont * ageGroup	f1	0.092	0.765	0.005
	f2	0.681	0.420	0.035		f2	1.407	0.250	0.069
	f3	1.045	0.320	0.052		f3	1.483	0.238	0.072
	f4	0.999	0.330	0.050		f4	1.363	0.257	0.067
	f5	1.279	0.272	0.063		f5	1.556	0.227	0.076
	f6	1.201	0.287	0.059		f6	1.307	0.267	0.064
	f7	0.887	0.358	0.045		f7	0.972	0.337	0.049
	f8	0.189	0.668	0.010		f8	0.209	0.653	0.011
anteriority	f1	5.928	0.025	0.238	anteriority	f1	4.908	0.039	0.205
	f2	7.385	0.014	0.280		f2	6.710	0.018	0.261
	f3	10.718	0.004	0.361		f3	10.282	0.005	0.351
	f4	12.880	0.002	0.404		f4	12.693	0.002	0.400
	f5	12.391	0.002	0.395		f5	12.424	0.002	0.395
	f6	12.730	0.002	0.401		f6	12.864	0.002	0.404
	f7	12.338	0.002	0.394		f7	12.590	0.002	0.399
	f8	71.990	0.000	0.791		f8	74.329	0.000	0.796
anteriority * ageGroup	f1	0.049	0.828	0.003	anteriority * ageGroup	f1	0.321	0.577	0.017
	f2	0.041	0.842	0.002		f2	0.008	0.929	0.000
	f3	0.001	0.980	0.000		f3	0.001	0.974	0.000
	f4	0.100	0.756	0.005		f4	0.078	0.783	0.004
	f5	0.153	0.700	0.008		f5	0.116	0.737	0.006
	f6	0.088	0.770	0.005		f6	0.059	0.811	0.003
	f7	0.069	0.796	0.004		f7	0.089	0.769	0.005
	f8	0.709	0.410	0.036		f8	0.744	0.399	0.038
ipsiCont * anteriority	f1	1.320	0.265	0.065	ipsiCont * anteriority	f1	0.601	0.448	0.031
	f2	0.013	0.910	0.001		f2	0.213	0.650	0.011
	f3	0.165	0.689	0.009		f3	0.302	0.589	0.016
	f4	0.426	0.522	0.022		f4	0.504	0.486	0.026
	f5	0.158	0.695	0.008		f5	0.189	0.669	0.010
	f6	0.039	0.845	0.002		f6	0.047	0.831	0.002
	f7	0.009	0.926	0.000		f7	0.004	0.949	0.000
	f8	0.412	0.529	0.021		f8	0.371	0.550	0.019
ipsiCont * anteriority * ageGroup	f1	1.466	0.241	0.072	ipsiCont * anteriority * ageGroup	f1	1.187	0.290	0.059
	f2	0.713	0.409	0.036		f2	0.262	0.614	0.014
	f3	0.012	0.915	0.001		f3	0.010	0.923	0.001
	f4	0.271	0.609	0.014		f4	0.379	0.545	0.020
	f5	0.851	0.368	0.043		f5	0.959	0.340	0.048
	f6	1.455	0.243	0.071		f6	1.536	0.230	0.075
	f7	0.880	0.360	0.044		f7	0.906	0.353	0.046
	f8	0.393	0.538	0.020		f8	0.466	0.503	0.024

Table 5: `Experiment 2 - table supplement 5:` Results of the repeated measures ANOVA with three within-subjects factors for the frequency decomposed SAM. f1 to f8 refer to the subbands in descending order of frequency. ipsiCont: ipsilateral vs. contralateral channel (with respect to the attended side). ageGroup: older vs. younger adults. anteriority: stimulus presentation from the front or back speakers. The left columns show the results when only the corresponding channel was used to reject artifacts, while the right columns display the results when artifact rejection additionally considers the M.SCM and HEOG.
